# Creation and characterization of novel knock-in mouse models of hypophosphatasia

**DOI:** 10.1093/jbmrpl/ziag147

**Published:** 2026-08-25

**Authors:** Rena Okawa, Marin Ochiai, Hiroshi Kurosaka, Tamami Kadota, Misato Takagi, Yuto Suehiro, Hiroko Okawa, Shuhei Naka, Satoshi Yamaguchi, Shin-Ichi Horike, Takiko Daikoku, Atsushi Watanabe, Michiyo Matsumoto-Nakano, Satoshi Imazato, Toshimi Michigami, Keiichi Ozono, Takashi Yamashiro, Kazuhiko Nakano

**Affiliations:** Department of Pediatric Dentistry, Graduate School of Dentistry, The University of Osaka, Suita 565-0871, Japan; Department of Pediatric Dentistry, Graduate School of Dentistry, The University of Osaka, Suita 565-0871, Japan; Department of Orthodontics and Dentofacial Orthopedics, Graduate School of Dentistry, The University of Osaka, Suita 565-0871, Japan; Department of Pediatric Dentistry, Graduate School of Dentistry, The University of Osaka, Suita 565-0871, Japan; Department of Pediatric Dentistry, Graduate School of Dentistry, The University of Osaka, Suita 565-0871, Japan; Department of Pediatric Dentistry, Graduate School of Dentistry, The University of Osaka, Suita 565-0871, Japan; Division of Molecular and Regenerative Prosthodontics, Tohoku University Graduate School of Dentistry, Sendai 980-8575, Japan; Department of Pediatric Dentistry, Okayama University Graduate School of Medicine, Dentistry and Pharmaceutical Sciences, Okayama 700-8558, Japan; Department of Dental Biomaterials, Graduate School of Dentistry, The University of Osaka, Suita 565-0871, Japan; Division of Integrated Omics Research, Research Center for Experimental Modeling of Human Disease, Kanazawa University, Kanazawa 920-8640, Japan; Division of Animal Disease Model, Research Center for Experimental Modeling of Human Disease, Kanazawa University, Kanazawa 920-8640, Japan; Division of Clinical Genetics, Kanazawa University Hospital, Kanazawa 920-8640, Japan; Department of Pediatric Dentistry, Okayama University Graduate School of Medicine, Dentistry and Pharmaceutical Sciences, Okayama 700-8558, Japan; Department of Dental Biomaterials, Graduate School of Dentistry, The University of Osaka, Suita 565-0871, Japan; Department of Bone and Mineral Research, Research Institute, Osaka Women’s and Children’s Hospital, Izumi 594-1101, Japan; Center for Promoting Treatment of Intractable Diseases, ISEIKAI International General Hospital, Osaka 530-0052, Japan; Department of Orthodontics and Dentofacial Orthopedics, Graduate School of Dentistry, The University of Osaka, Suita 565-0871, Japan; Department of Pediatric Dentistry, Graduate School of Dentistry, The University of Osaka, Suita 565-0871, Japan

**Keywords:** hypophosphatasia, alkaline phosphatase, cementum, early exfoliation of primary teeth, hypomineralization

## Abstract

Hypophosphatasia (HPP) is a rare inherited disorder caused by inactivating variants in the *ALPL* gene, resulting in defective mineralization of bone and teeth. Dental manifestations include early exfoliation of fully rooted primary teeth as a result of cementum dysplasia, premature loss of permanent teeth, and hypomineralization of enamel, dentin, and jawbone. However, genotype-dependent dental phenotypes and severity-based treatment strategies remain poorly defined. The aim of this study was to generate knock-in mouse models harboring clinically relevant *ALPL* variants and to characterize their dental and periodontal phenotypes in relation to serum tissue-nonspecific alkaline phosphatase (TNAP) activity. We analyzed mice that were compound heterozygous for a null variant and a hypomorphic variant (*Alpl^c.1559delT/p.F327L^*) and mice that were heterozygous for a dominant-negative variant (*Alpl^+/p.R184W^*). Dental and alveolar phenotypes were assessed using micro-CT, histological analyses, and periodontal ligament traction experiments. *Alpl^c.1559delT/p.F327L^* mice exhibited reduced serum TNAP levels, mild skeletal abnormalities, and severe dental defects, including hypomineralization of enamel, dentin, and cementum, decreased alveolar BMD, and an increased distance from the cemento–enamel junction to the alveolar bone crest. In contrast, *Alpl^+/p.R184W^* mice showed isolated dental abnormalities characterized by dentin and cementum hypomineralization and decreased alveolar bone BMD without overt skeletal involvement. Periodontal tissue fragility was increased in both models. These knock-in mouse models recapitulate genotype-dependent dental manifestations of HPP and indicate that dental phenotypes are influenced not only by circulating TNAP levels but also by the functional effects of individual *ALPL* variants. This platform provides a valuable framework for elucidating the mechanisms underlying genotype-dependent dental manifestations and for developing dental treatment strategies tailored to disease severity.

## Introduction

Hypophosphatasia (HPP) is a rare inherited skeletal disease caused by pathogenic variants of the *ALPL* gene.^1^ These variants exhibit a reduction in tissue-nonspecific alkaline phosphatase (TNAP) enzymatic activity, which impairs bone and tooth mineralization.^1–4^ Hypophosphatasia is currently classified into 6 phenotypes: perinatal severe, perinatal benign, infantile, childhood, adult, and odontohypophosphatasia (odonto).^1–4^ A typical dental symptom of HPP is an early exfoliation of primary teeth as a result of cementum dysplasia caused by low serum TNAP level.^5^ This dental manifestation sometimes leads to a diagnosis of a milder form of HPP, such as odonto or childhood HPP.^6^ Tooth fractures, severe caries, an increased risk of periodontitis, and early loss of permanent teeth have also been reported in adult HPP.^7^ The more severe forms of HPP have been shown to be associated with enamel and dentin hypomineralization, malocclusion, impaired oral function, and dysphagia, in addition to early exfoliation of primary teeth.^8^ Although HPP patients require multidisciplinary dental treatment, including prosthodontic rehabilitation, periodontal management, orthodontic treatment, and implant therapy depending on disease severity, no standardized severity-based dental treatment strategy has been established. The development of fundamental treatments to prevent tooth loss, in addition to elucidating the effects of enzyme replacement therapy (ERT) on the jawbone during orthodontic treatment and implant placement, is urgently required.^5^^,^^6^^,^^9^ In addition to systemic skeletal manifestations, dental and periodontal abnormalities are increasingly recognized as major contributors to morbidity and reduced quality of life in patients with HPP.

The more severe forms of HPP (perinatal severe and infantile forms) usually follow an autosomal recessive form of inheritance, with variants in both alleles.^2^ However, in milder forms of HPP (childhood, adult, and odonto HPP), some families have an autosomal recessive form of inheritance, and others exhibit an autosomal dominant form of inheritance. The prevalence of HPP is estimated at 1:100 000-300 000 for severe forms.^10^ Moderate and mild HPP are considered to be underdiagnosed.^9^ In Europe, the prevalence of moderate and mild HPP has been estimated to be approximately 1:2430 and 1:508, respectively.^11^ Genetic and dental manifestations of patients with odonto and non-odonto HPP are different, and these differences should be considered during clinical treatment of patients with HPP.^8^^,^^12^

To date, more than 480 *ALPL* variants responsible for HPP have been identified and are summarized in the ALPL Gene Variant Database (https://alplmutationdatabase.jku.at/). Genotype–phenotype correlations in HPP are complex, with significant variability even among patients carrying identical *ALPL* variants. Nevertheless, several recurrent variants have been well characterized with respect to their clinical phenotypes, including a thymine deletion at nucleotide 1559 (c.1559delT), the p.F327L variant, which results from the substitution of phenylalanine with leucine at codon 327, and the p.R184W variant.^13–16^ The c.1559delT variant (p.Leu520ArgfsX86 in exon 12) results primarily in the loss of TNAP enzyme activity. Homozygosity for the c.1559delT variant is associated with perinatal severe HPP.^16^ p.F327L (c.979T>C, p.Phe327Leu in exon 9) leads to substantial residual enzyme activity, equivalent to approximately 70% of the WT protein, and is associated with perinatal benign HPP in compound heterozygous individuals.^13^ The heterozygous p.R184W variant (c.550C>T, p.Arg184Trp in exon 6) has been identified in patients with odonto HPP^3^^,^^13^^,^^16^ and exhibits a dominant-negative effect, resulting in markedly reduced TNAP enzymatic activity even in the heterozygous state.^17^ These 3 variants were selected, because they represent distinct clinical phenotypes across the HPP spectrum, enabling investigation of genotype-dependent dental phenotypes across different severities of HPP.

We hypothesized that mice that are homozygous for the c.1559delT (*Alpl^c.1559delT/c.1559delT^*) variant would phenocopy the more severe HPP phenotypes, while mice with the c.1559delT/p.F327L (*Alpl^c.1559delT/p.F327L^*) genotype would model mild forms of HPP, and mice heterozygous for the p.R184W (*Alpl^+/p.R184W^*) variant would represent odontohypophosphatasia, the mildest clinical form of HPP.

The aim of this study was to create knock-in (KI) mice with different pathogenic variants of the *ALPL* gene and analyze their phenotypes, with a particular focus on dental characteristics, such as the alveolar bone, tooth structure, and the periodontal ligament (PDL).

## Materials and methods

### Ethics statement

All animal studies were approved by the IACUC of Kanazawa University and Osaka University Graduate School of Dentistry (permit number AP-204127, R-03-007-0). All experimental protocols were approved by the Gene Modification Experiments Safety Committee of Osaka University (permit number 04795).

### Generation of KI mice

A CRISPR/Cas9-mediated targeting strategy generated *Alpl* p.R184W, p.F327L, and c.1559delT KI mice. Additional details are provided in the supplementary information and Table S1.

Heterozygous KI mice carrying the c.1559delT or p.F327L variant were crossed with heterozygous KI mice carrying either the same or a different variant. Heterozygous KI male mice carrying the p.R184W variant were crossed with WT female mice. Tail samples were obtained from offspring at the age of 3 wk, and genomic DNA was extracted for genotyping (Figure S1). Primary analyses of systemic skeletal phenotypes, including body weight, serum TNAP activity, and long-bone measurements, were performed using 12-wk-old male mice. Corresponding analyses were also performed in female mice. Dental analyses, including morphometric and histological evaluations, were performed using both male and female mice.

### Measurement of serum TNAP

WT (*n* = 15 males), *Alpl^+/c.1559delT^* (*n* = 4 males), *Alpl^+/p.F327L^* (*n* = 7 males), *Alpl^+/p.R184W^* (*n* = 8 males), and *Alpl^c.1559delT/p.F327L^* (*n* = 4 males) mice were anesthetized with sodium pentobarbital and weighed at 12 wk after birth. Blood samples were obtained from the heart. The blood serum was collected using centrifugation at 16 100 × *g* for 15 min, and serum TNAP values were measured by BML, Inc.

### Tissue preparation

WT and KI mice were fixed by transcardiac perfusion with 4% paraformaldehyde under general anesthesia at 12 wk after birth. The mandibles were removed from the crania and cut into hemisections. After perfusion fixation, the tibiae were dissected, and the tibial length and width were measured using a digital caliper to assess gross skeletal growth. Tibial length was measured from the medial condyle to the medial malleolus, and tibial width was measured as the maximum mediolateral diameter at the widest point of the proximal tibial epiphysis. One mandibular hemisection and long bones were post-fixed in 4% paraformaldehyde overnight at 4 °C for CT and histological examination. Other mandibular hemisections for PDL traction experiments were immersed in PBS and subjected to the traction test immediately.

### Micro-CT analysis

The long hind limb bones (femurs and tibiae; WT: *n* = 10-15 males, *Alpl^+/p.R184W^*: *n* = 7-8 males, *Alpl^c.1559delT/p.F327L^*: *n* = 4 males) and mandibular hemisections were scanned using a small-animal CT system (R_mCT2; Rigaku) at 90 kVp, 160 μA, 180 s integration time, and 20-μm voxel dimension. Alveolar bone BMD analyses were performed using WT (*n* = 15 males), *Alpl^+/p.R184W^* (*n* = 7 males), and *Alpl^c.1559delT/p.F327L^* (*n* = 4 males) mice. Mandibular tooth morphometric analyses were performed using WT (*n* = 19; males and females), *Alpl^+/p.R184W^* (n = 16; males and females), and *Alpl^c.1559delT/p.F327L^* (*n* = 12; males and females) mice. Enamel and dentin mineral density (MD) analyses were performed using WT (*n* = 26; both males and females), *Alpl^+/p.R184W^* (*n* = 17; both males and females), and *Alpl^c.1559delT/p.F327L^* (*n* = 11; both males and females) mice. Mineral density measurements were calibrated using the manufacturer’s hydroxyapatite phantom. Images were reconstructed and analyzed at the original acquisition resolution without downsizing. BMD values for the trabecular and cortical compartments of the long bones and mandibular bone, and MD values for enamel and dentin were calculated using 3D trabecular structure analysis software (TRI/3D-BON; RATOC System Engineering Co.). For long bone analysis, ROIs were defined in the metaphyseal regions adjacent to the growth plates. In the femur, the ROI was located 1.0 mm proximal to the distal growth plate and extended over a width of 1.0 mm. In the tibia, the ROI was located 1.0 mm distal to the proximal growth plate and extended over a width of 1.0 mm. For mandibular analysis, alveolar bone BMD was calculated as the average of measurements obtained from 3 anatomical sites: the interradicular bone of the first molar, the interdental bone between the first and second molars, and the mesial alveolar bone of the first molar. The BMD of inferior cortical bone beneath the first molar was assessed separately. For tooth analysis, MD was measured at predefined ROIs in the enamel and dentin of sagittal sections through the mandibular first molar at the level of the maximum mesiodistal width (Figure S2A). Mineral density values were calculated using TRI/3D-BON software based on calibrated grayscale values. Trabecular microarchitectural parameters, including bone volume fraction (BV/TV) and trabecular thickness (Tb.Th), were calculated using the same software, with Tb.Th derived using a model-independent sphere-fitting method based on a distance transformation algorithm (*n* = 5 males per group). The width of the enamel was defined as the horizontal distance from the maximum bulge to the enamel–dentin junction in the image of the first molar’s crown width (Figure S2B). Similarly, the width of the dentin was defined as the horizontal distance from the enamel–dentin junction to the pulp chamber. The mandibular first molar was isolated from the sagittal CT images (Figure S2C and D), and the pulp cavity was segmented from the isolated tooth (Figure S2E). The areas of the tooth and segmented pulp cavity were measured using a Java-based image processing program (ImageJ, NIH), and the percentage of the pulp cavity area relative to the total tooth area was calculated. Alveolar bone loss was assessed at 2 sites (distobuccal cusp and distal cusp) of the first molar, 3 sites (mesiobuccal cusp, buccal cusp, and distobuccal cusp) of the second molar, and 1 site (buccal cusp) of the third molar by measuring the distance from the cemento–enamel junction (CEJ) to the alveolar bone crest (ABC) on the buccal and lingual side of the alveolar bone (*n* = 5 mice [2 males, 3 females] per group).^18^ Total bone loss was calculated from the 12-site total CEJ–ABC distance.

### Histological examination

Other mandibular hemisections (*n* = 5 mice [2 males, 3 females] per group) were demineralized with 10% EDTA in 0.1 M Tris pH 7.0 for 3 wk at 4 °C with gentle agitation, with the EDTA solution changed every 3 d, and then conventionally processed for paraffin embedding. The mandibular hemisections were cut into 5-μm-thick cross-sections through the incisor at the level of the mesial root of the first molar and sagittal sections through the first molar, mounted on silane-coated slides, stained with H&E, and analyzed immunohistochemically. Antigens were retrieved with 0.1 M citrate buffer pH 6.0 at 70 °C. For immunohistochemical analysis of osteopontin (OPN; a protein expressed in cementum and bone), a polyclonal rabbit anti-mouse OPN antibody (Immuno-Biological Laboratories Co., Ltd.) was used as the primary antibody at a dilution of 1:500. The bound primary antibody was detected with a horseradish peroxidase-labeled anti-rabbit secondary antibody (Thermo Fisher Scientific) and visualized with diaminobenzidine as the substrate. The sections were then counterstained with hematoxylin. The distance from the CEJ to the apex of the mesial root of the first molar was defined as 100%. The cumulative length of the OPN-positive regions along the root surface was measured and expressed as a percentage of the total root length. Osteoclasts were identified as tartrate-resistant acid phosphatase (TRAP)-positive multinucleated cells using a TRAP staining kit (FUJIFILM Wako Pure Chemical Corporation). To quantify osteoclasts, the alveolar bone perimeter facing the first molar was outlined using the freehand selection tool in ImageJ (NIH). The number of TRAP-positive multinucleated osteoclasts was manually counted and expressed as the number of osteoclasts per bone perimeter (N.Oc/B.Pm, cells/mm).

### PDL traction experiments

To assess the strength of the PDL, a model was created in which the PDL was physically torn off during tooth extraction as described by Trombetta-eSilva et al. (*n* = 5 mice [2 males, 3 females] per group) (Figure S3A-E).^19^ A 0.2-mm diameter tungsten wire (Niraco) was threaded through the first molar root bifurcation. Mandibular hemisections were reinforced with resin composite (Clearfil Majesty ES Flow, Kuraray Noritake Dental). The resin-reinforced mandibular hemisection was secured in a universal testing machine (EZ-S 500N; Shimadzu), and the tungsten wire was pulled at a crosshead speed of 1 mm/min. The maximum load applied before the first molar was dislodged from its socket was measured.

### Statistical analysis

Statistical analyses were conducted using GraphPad Prism 10 Statistics Software (GraphPad Software Inc.). Data are shown as the mean ± SEM, except for the sex-stratified analyses in Figure S9, which are shown as the mean ± SD. Outliers in the serum TNAP data were identified and excluded using Thompson’s rejection test according to a predefined criterion. One-way ANOVA followed by Dunnett’s multiple comparisons test was used to compare each KI group with the WT group. For enamel and dentin widths, the pulp-to-tooth ratio, and enamel and dentin MD, one-way ANOVA followed by Bonferroni’s multiple comparisons test was used for pairwise comparisons among the 3 groups. *p-*values of less than .05 were considered statistically significant.

## Results

### *Alpl* variants reduced serum TNAP level and mildly affected skeletal growth

**Figure 1:**
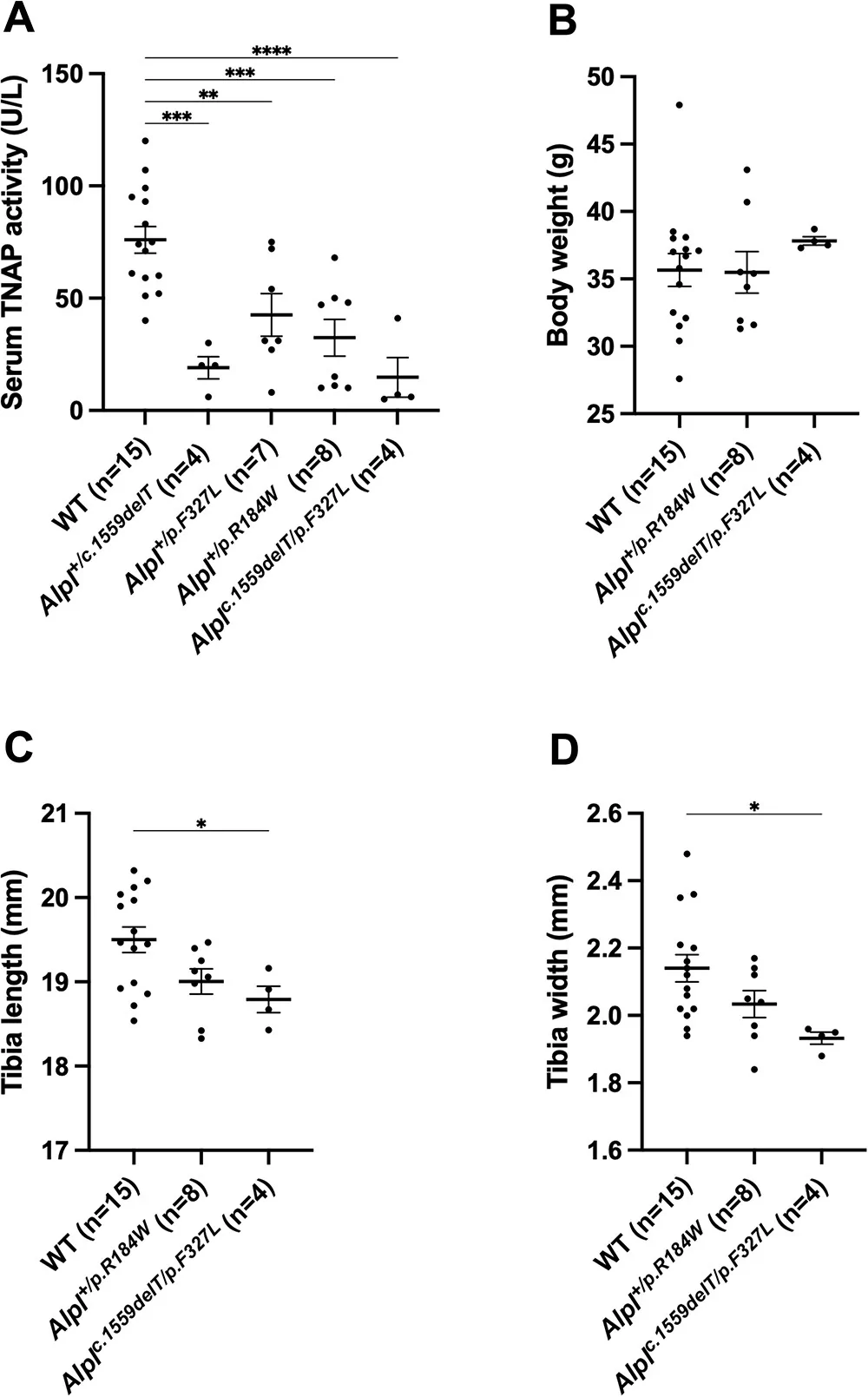
General characteristics of WT and knock-in (KI) mice. (A) Distribution of serum tissue-nonspecific alkaline phosphatase (TNAP) activity. (B) Body weight. (C) Tibial length. (D) Tibial width. Data represent the mean ± SEM. Significant differences were determined using one-way ANOVA followed by Dunnett’s multiple comparisons test. WT (*n* = 15 males), *Alpl^+/c.1559delT^* (*n* = 4 males), *Alpl^+/p.F327L^* (*n* = 7 males), *Alpl^+/p.R184W^* (*n* = 8 males), and *Alpl^c.1559delT/p.F327L^* (*n* = 4 males). ^*^p < .05, ^**^p < .01, ^***^p < .001, and ^****^p < .0001.

No *Alpl^c.1559delT/c.1559delT^* mice were detected among the surviving pups at the time of genotyping (3 wk after birth). No significant differences in survival to weaning at 3 wk postnatal were observed among WT, *Alpl^+/p.R184W^*, and *Alpl^c.1559delT/p.F327L^* mice (data not shown). Serum TNAP level was significantly reduced in *Alpl^+/c.1559delT^* (*p* = .0003), *Alpl^+/p.F327L^* (*p* = .0090), *Alpl^+/p.R184W^* (*p* = .0003), and *Alpl^c.1559delT/p.F327L^* (*p* < .0001) mice compared to WT mice (Figure 1A). Female mice showed a similar trend in serum TNAP activity (Figure S4A). Outliers in the serum TNAP data were excluded using Thompson’s rejection test according to the predefined criterion. The remaining variability may reflect biological variation among heterozygous individuals. Body weight at 12 wk of age did not differ significantly between any KI group and the WT group (*p* > .05) (Figure 1B). The tibiae were significantly shorter and slimmer in *Alpl^c.1559delT/p.F327L^* mice compared to WT mice (*p* = .0423 and 0.0230, respectively) (Figure 1C and D). Female mice showed similar overall trends, although the differences were less pronounced than those observed in male mice (Figure S4B-D). As expected for carrier states, *Alpl^+/c.1559delT^* and *Alpl^+/p.F327L^* mice showed no overt skeletal phenotype, with normal body weight and tibial length and width (Figure S5), and were therefore not included in the main analysis.

### BMD of long bones was largely preserved, whereas alveolar bone density was reduced in *Alpl* mutant mice

**Figure 2:**
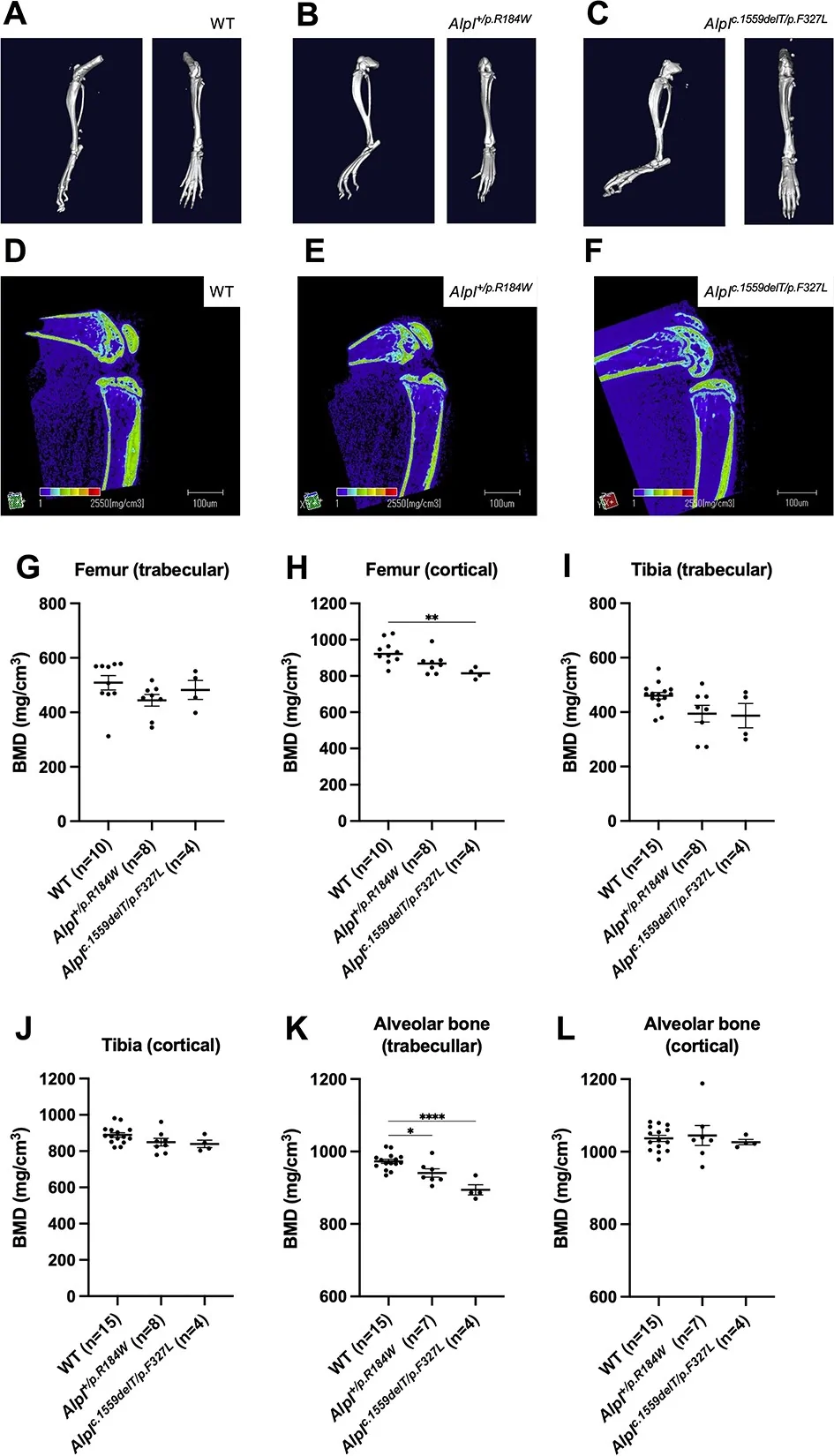
Micro-CT analysis and BMD of long bones and mandibles in WT and knock-in (KI) mice. (A-C) Representative 3-dimensional micro-CT images of the hind limb bones in lateral and frontal views from (A) WT mice, (B) *Alpl^+/p.R184W^* mice, and (C) *Alpl^c.1559delT/p.F327L^* mice. (D-F) Representative color-coded visualizations of BMD in the distal femur and proximal tibia from (D) WT mice, (E) *Alpl^+/p.R184W^* mice, and (F) *Alpl^c.1559delT/p.F327L^* mice. Femoral BMD: (G) trabecular and (H) cortical components. WT (*n* = 10 males), *Alpl^+/p.R184W^* (*n* = 8 males), and *Alpl^c.1559delT/p.F327L^* (*n* = 4 males). Tibial BMD: (I) trabecular and (J) cortical components. WT (*n* = 15 males), *Alpl^+/p.R184W^* (*n* = 8 males), and *Alpl^c.1559delT/p.F327L^* (*n* = 4 males). Alveolar bone BMD: (K) trabecular and (L) cortical components. WT (*n* = 15 males), *Alpl^+/p.R184W^* (*n* = 7 males), and *Alpl^c.1559delT/p.F327L^* (*n* = 4 males). Data represent the mean ± SEM. Significant differences were determined using one-way ANOVA followed by Dunnett’s multiple comparisons test. ^*^p < .05, ^**^p < .01, and ^****^p < .0001.

Figure 2A-F show representative micro-CT images of hind limb bones from the mice in each group. There was no apparent difference in bone morphology between genotypes. BMD values for the long bones and alveolar bone are shown in Figure 2G-L. Trabecular BMD of the femur and tibia and cortical BMD of the tibia did not differ significantly among genotypes (*p* > .05); however, the BMD of the cortical bone of the femur in *Alpl^c.1559delT/p.F327L^* mice was significantly lower than that in WT mice (*p* = .0049) (Figure 2G-J). BMD measurements in female mice also showed similar trends (Figure S4E-H). No apparent differences were observed in the qualitative color-coded BMD visualizations of the long bones (Figure 2D-F). Consistent with these findings, trabecular microarchitectural parameters, including BV/TV and Tb.Th, showed no significant differences among the genotypes in either the femur or tibia (Figure S6A-D). Trabecular alveolar bone BMD was significantly lower in *Alpl^+/p.R184W^* (*p* = .0210) and *Alpl^c.1559delT/p.F327L^* mice (*p* < .0001) than in WT mice, whereas cortical alveolar bone BMD did not differ significantly among the genotypes (Figure 2K and L). Consistent with these findings, BV/TV was reduced in *Alpl^+/p.R184W^* and *Alpl^c.1559delT/p.F327L^* mice compared with WT mice, whereas Tb.Th did not differ significantly among the genotypes (Figure S6E and F).

### Micro-CT analysis revealed genotype-dependent tooth and alveolar bone mineralization defects

**Figure 3:**
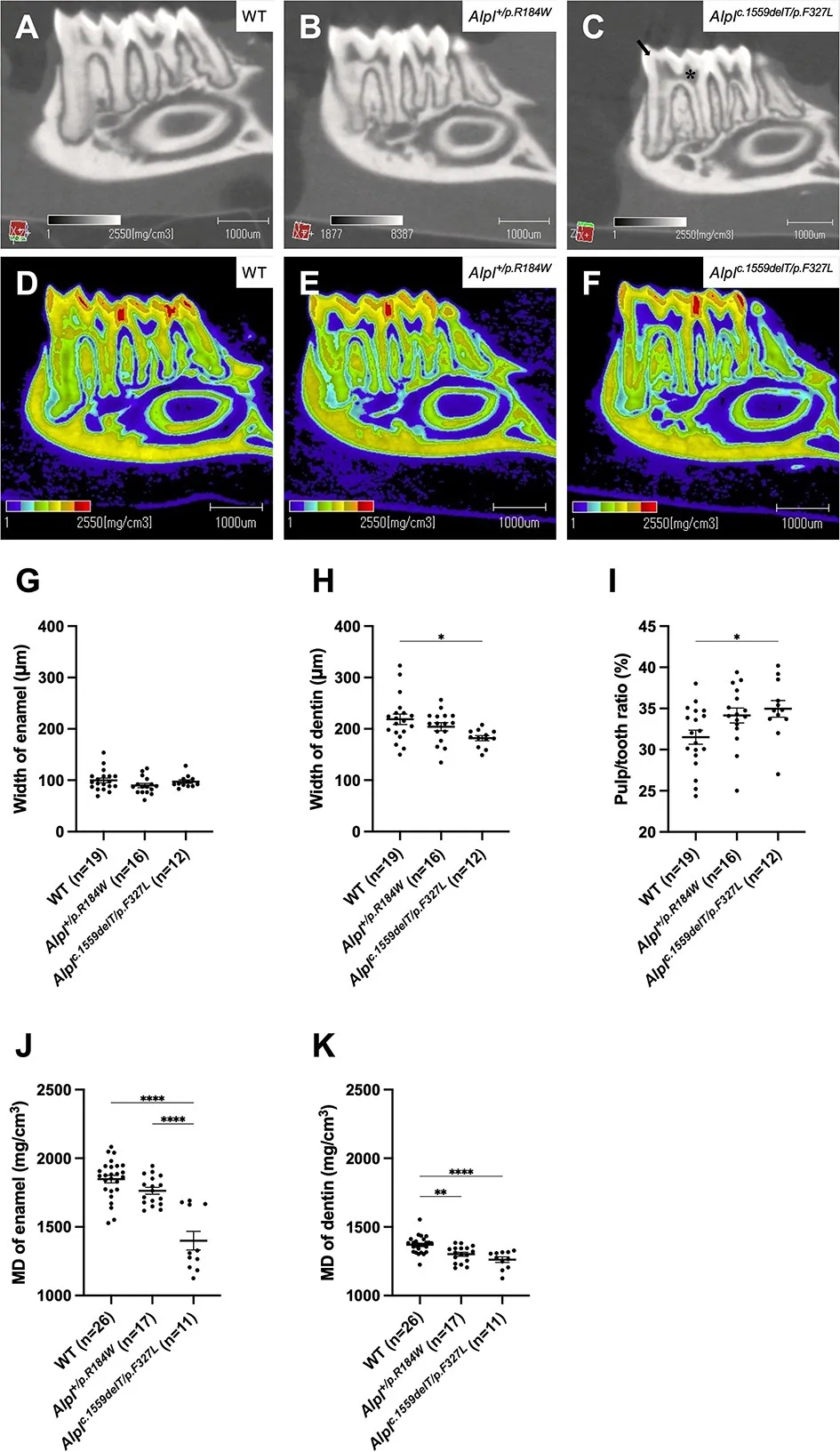
Micro-CT analysis of mandibles from WT and knock-in (KI) mice. (A-C) Sagittal images of representative mandibles from (A) WT mice, (B) *Alpl*^+*/p.R184W*^ mice, and (C) *Alpl^c.1559delT/p.F327L^* mice. Arrow: thin dentin; asterisk: enlarged pulp chamber. (D-F) Visualization of BMD in images of representative mandibles from (D) WT mice, (E) *Alpl^+/p.R184W^* mice, and (F) *Alpl^c.1559delT/p.F327L^* mice. (G and H) Tooth width: (G) enamel and (H) dentin. (I) Pulp/tooth ratio of the first molar. WT (*n* = 19; both males and females), *Alpl^+/p.R184W^* (*n* = 16; both males and females), and *Alpl^c.1559delT/p.F327L^* (*n* = 12; both males and females). (J and K) Mineral density (MD) of tooth: (J) enamel and (K) dentin. WT (*n* = 26; both males and females), *Alpl^+/p.R184W^* (*n* = 17; both males and females), and *Alpl^c.1559delT/p.F327L^* (*n* = 11; both males and females). Data represent the mean ± SEM. Significant differences were determined using one-way ANOVA followed by Bonferroni’s multiple comparisons test. ^*^p < .05, ^**^p < .01, and ^****^p < .0001.

*Alpl^c.1559delT/p.F327L^* mice showed abnormal dentin morphology characterized by thin dentin and enlarged pulp chambers when compared with WT and *Alpl^+/p.R184W^* mice (Figure 3A-C, Figure S7A-C). Enamel width did not differ significantly between genotypes (*p* > .05); however, dentin width in *Alpl^c.1559delT/p.F327L^* mice was significantly smaller than that in WT mice (*p* = .0196) (Figure 3G and H). The pulp/tooth ratio of *Alpl^c.1559delT/p.F327L^* mice was significantly higher than that of WT mice (*p* = .0411); however, no significant differences were observed between the *Alpl^+/p.R184W^* mice and the WT mice (Figure 3I). The MD of enamel was significantly lower in *Alpl^c.1559delT/p.F327L^* mice compared to WT mice and *Alpl^+/p.R184W^* mice (*p* < .0001) (Figure 3J). The MD of dentin was significantly lower in *Alpl^+/p.R184W^* mice (*p* = .0013) and *Alpl^c.1559delT/p.F327L^* (*p* < .0001) mice compared to WT mice (Figure 3K). Similar trends were observed in both sexes across all dental parameters, with no apparent sex-specific differences (Figure S8).

**Figure 4:**
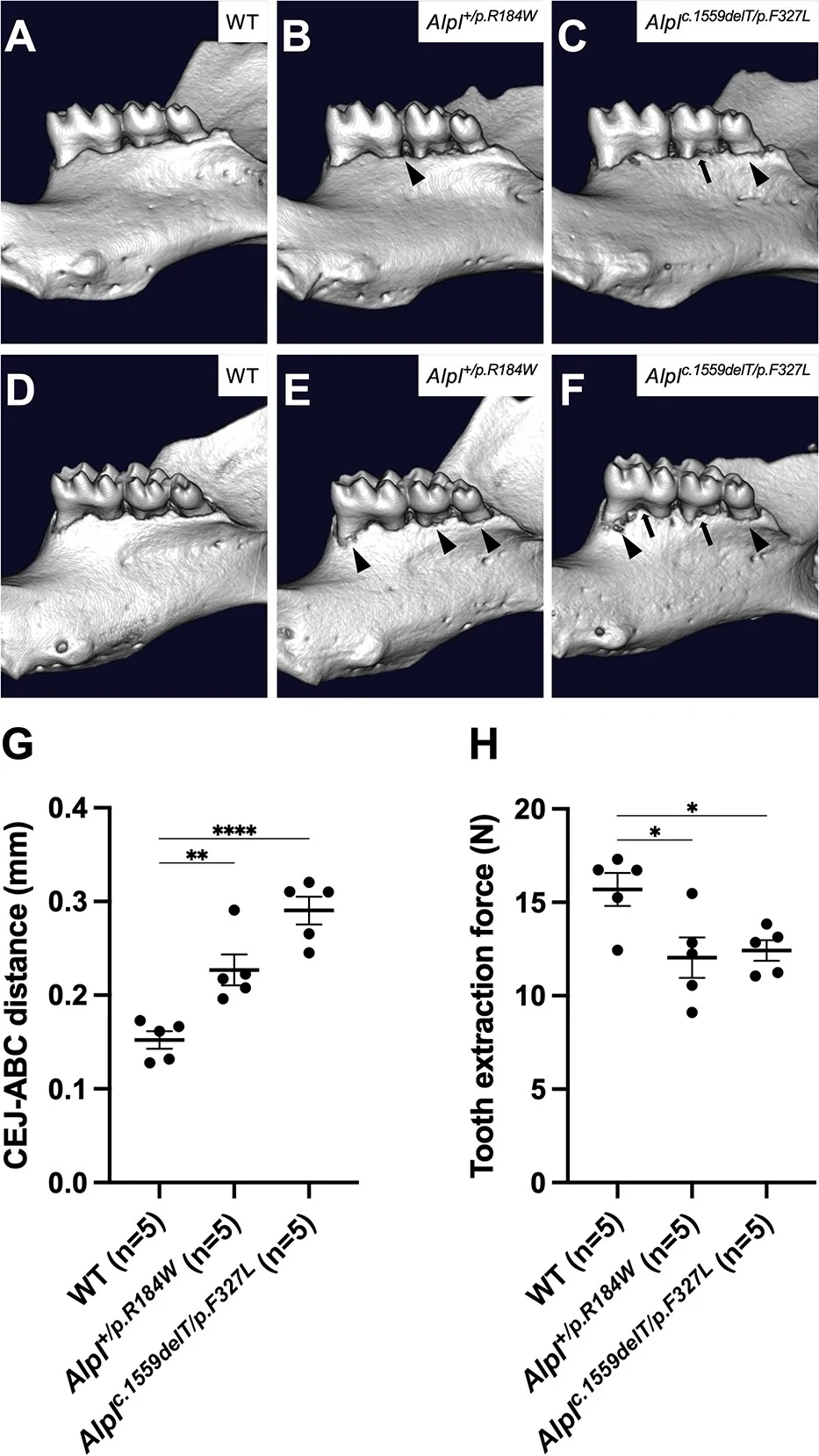
Three-dimensional micro-CT analysis of alveolar bone and periodontal ligament (PDL) traction testing in WT and knock-in (KI) mice. (A and D) WT mice, (B and E) *Alpl^+/p.R184W^* mice, and (C and F) *Alpl^c.1559delT/p.F327L^* mice. (A-C) Buccal and (D-F) lingual views. Arrowheads: reduced alveolar bone height; arrow: exposed root bifurcation of the first molar. (G) Alveolar bone loss was measured at the molar from the cemento–enamel junction (CEJ) to the alveolar bone crest (ABC). (H) Periodontal ligament traction test with tooth extraction. Statistical analyses were performed using one-way ANOVA followed by Dunnett’s multiple comparisons test. Data are represented as mean ± SEM (*n* = 5 mice [2 males and 3 females] per group). ^*^p < .05, ^**^p < .01, and ^****^p < .0001.

Figure 4A-F show representative micro-CT images of the mandibles of the mice in each group. Reduced alveolar bone height around the root of the molar was observed in *Alpl^+/p.R184W^* (Figure 4B and E) and *Alpl^c.1559delT/p.F327L^* mice (Figure 4C and F). The root bifurcation of the first molar in *Alpl^c.1559delT/p.F327L^* mice was exposed. The CEJ–ABC distance was significantly increased in *Alpl^+/p.R184W^* (*p* = .0074) and *Alpl^c.1559delT/p.F327L^* (*p* < .0001) mice compared with WT mice (Figure 4G). A similar trend in alveolar bone loss was observed in both sexes, with no apparent sex-specific differences (Figure S9A).

### Histological, immunohistochemical, and functional analyses revealed periodontal tissue fragility in *Alpl* mutant mice

To investigate the strength of the PDL, the maximum load required for tooth extraction was determined (Figure S3A-E). The first molar was completely dislocated and no root fracture was observed (Figure S3F and G). The maximum load during tooth extraction via PDL traction in *Alpl^+/p.R184W^* mice and *Alpl^c.1559delT/p.F327L^* mice was significantly lower than that in WT mice (*p* = .0209 and *p* = .0368, respectively) (Figure 4H). A similar trend in the maximum load during tooth extraction was observed in both sexes, with no apparent sex-specific difference (Figure S9B).

**Figure 5:**
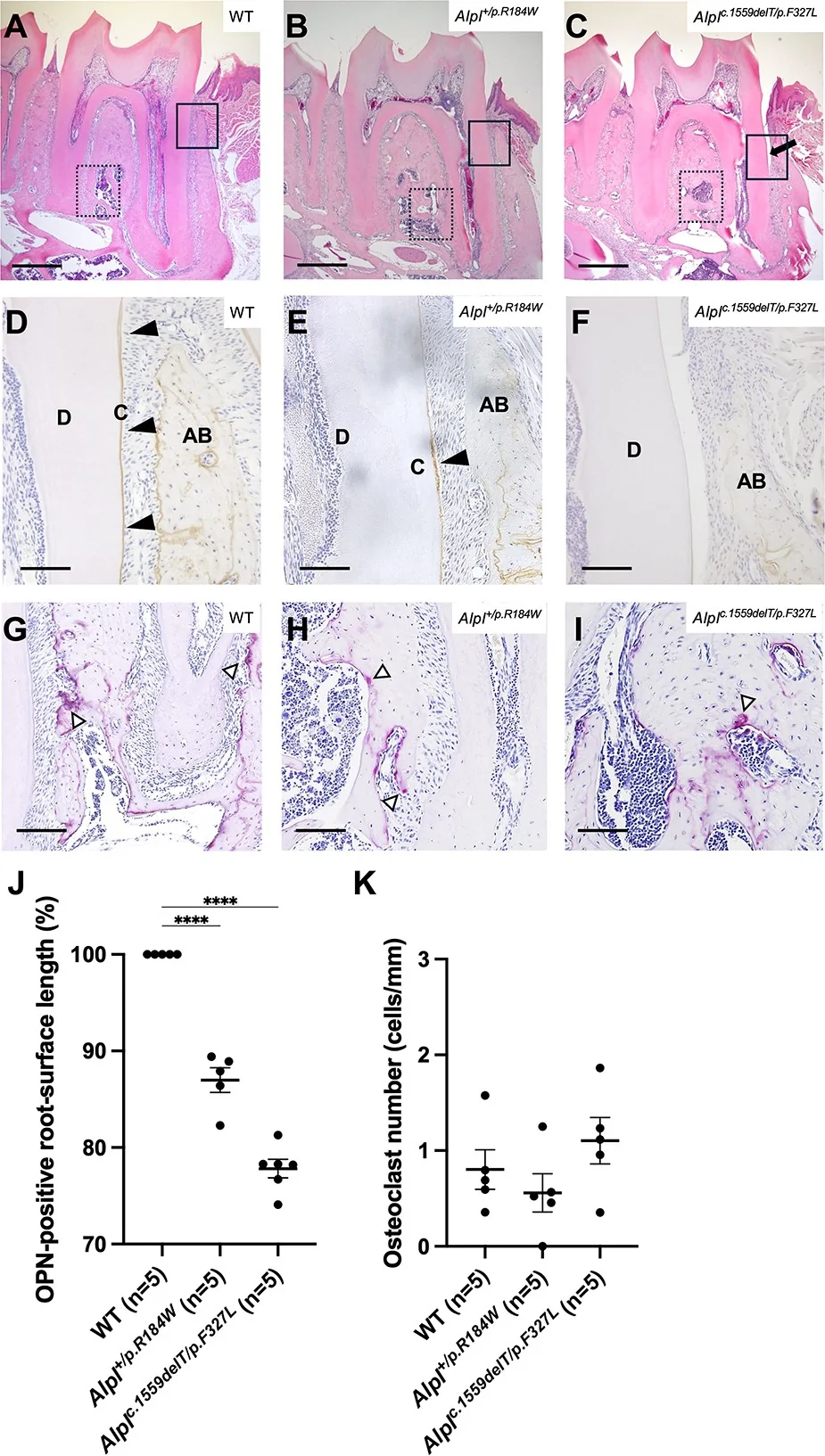
Histological analysis in WT and knock-in (KI) mice. (A-C) H&E staining. (D-F) Immunohistochemical analysis of osteopontin (OPN). (G-I) Tartrate-resistant acid phosphatase (TRAP) staining. Representative sagittal sections of the mandibular first molar are shown for: (A, D, and G) WT mice; (B, E, and H) *Alpl^+/p.R184W^* mice; and (C, F, and I) *Alpl^c.1559delT/p.F327L^* mice. (D-F) are higher magnification images of the squares in A-C. G-I are higher magnification images of the dotted squares in A-C. Scale bars: (A-C) 500 μm, (D-F) 100 μm, and (G-I) 200 μm. Arrow: detachment between the tooth root and gingiva, black arrowheads: osteopontin, and white arrowheads: TRAP-positive osteoclasts. AB, alveolar bone; C, cementum; D, dentin. (J) Osteopontin-positive root-surface length (%) along the mesial root of the first molar. Data are presented as mean ± SEM (*n* = 5 mice [2 males and 3 females] per group). Statistical analysis was performed using one-way ANOVA followed by Dunnett’s multiple comparisons test. ^****^p < .0001. (K) Quantification of TRAP-positive multinucleated osteoclasts on the alveolar bone surface facing the first molar (M1), expressed as osteoclast number per bone perimeter (N.Oc/B.Pm, cells/mm). Data are presented as mean ± SEM (*n* = 5 mice [2 males and 3 females] per group). Statistical analysis was performed using one-way ANOVA with Dunnett’s multiple comparisons test.

Figure 5 shows representative histological, immunohistochemical, and histochemical images of the first molar for each group of mice. No obvious histological abnormalities were observed in the dentin of *Alpl^+/p.R184W^* and *Alpl^c.1559delT/p.F327L^* mice compared with WT mice (Figure 5A-C). However, detachment between the tooth root and gingiva was detected in *Alpl^c.1559delT/p.F327L^* mice (Figure 5C). Osteopontin immunoreactivity was observed on the surface of the alveolar bone in all groups, although it appeared reduced in *Alpl^c.1559delT/p.F327L^* mice. On the root surface, OPN immunoreactivity was continuous in WT mice, discontinuous in *Alpl^+/p.R184W^* mice, and nearly absent in *Alpl^c.1559delT/p.F327L^* mice (Figure 5D-F). Tartrate-resistant acid phosphatase-positive osteoclasts were observed on the alveolar bone surface of WT, *Alpl^+/p.R184W^* and *Alpl^c.1559delT/p.F327L^* mice (Figure 5G-I). The OPN-positive root-surface length along the mesial root of the first molar was significantly decreased in *Alpl^+/p.R184W^* and *Alpl^c.1559delT/p.F327L^* mice compared with WT mice (*p* < .0001) (Figure 5J). In contrast, neither *Alpl^+/p.R184W^* nor *Alpl^c.1559delT/p.F327L^* mice showed a significant difference in the number of osteoclasts on the alveolar bone surface compared with WT mice (*p* > .05; Figure 5K). Figure S10 shows representative immunohistochemical and H&E-stained images of the incisor for each group of mice. Abnormal dentin formation was observed in *Alpl^c.1559delT/p.F327L^* mice (Figure S10C and F). Osteopontin was detected on the surface of the alveolar bone and root cementum in WT mice (Figure S10D and G). *Alpl^+/p.R184W^* mice showed weak and irregular OPN expression on the incisor root surfaces (Figure S10E and H). *Alpl^c.1559delT/p.F327L^* mice lacked OPN expression on the incisor root surfaces and showed weak OPN expression on the surface of alveolar bone (Figure S10F and I). Periodontal ligament fiber organization and attachment appeared comparable among the groups. However, *Alpl^c.1559delT/p.F327L^* mice exhibited a reduction in cellularity compared to WT and *Alpl^+/p.R184W^* mice (Figure S10G-I).

## Discussion

More than 480 *ALPL* gene variants have been reported in the ALPL Gene Variant Database (https://alplmutationdatabase.jku.at).^20^ c.1559delT and p.F327L variants are commonly found and are associated with severe and milder forms of HPP, respectively.^13^ The c.1559delT variant is frequently identified in patients with perinatal severe HPP and causes complete loss of TNAP enzymatic activity, whereas p.F327L retains partial enzymatic activity and is associated with milder phenotypes.^13–16^ In this study, we aimed to create a mouse model for the perinatal severe phenotype. However, *Alpl^c.1559delT/c.1559delT^* mice could not be identified because genotyping was performed 3 wk after birth. Therefore, we cannot exclude the possibility that homozygous mice were embryonic lethal or survived until birth and died before genotyping. If ERT is started immediately after delivery, the dental manifestations of severe forms and the dental effects of ERT could be elucidated.

Bone mineralization defects and premature loss of primary teeth are the primary symptoms of HPP and serve as diagnostic triggers.^21^ Therefore, serum TNAP levels and bone density were evaluated to determine whether the KI mice reproduce the biochemical and skeletal characteristics of the HPP subtypes associated with each *ALPL* variant. Carriers of pathogenic *ALPL* variants associated with autosomal recessive HPP are usually asymptomatic, although they often exhibit reduced serum TNAP levels.^5^^,^^16^ The p.R184W variant, which is associated with autosomal dominant inheritance, is frequently reported in patients with odonto HPP.^4^^,^^12^^,^^21^ Odonto HPP is the mildest form, presenting without systemic symptoms.^3^^,^^12^^,^^15^ In this study, serum TNAP levels were significantly decreased in *Alpl^+/p.R184W^* mice compared with those in WT mice, and no overt skeletal phenotype was observed. The p.F327L variant retains substantial residual function and is associated with milder HPP phenotypes, and the c.1559delT/p.F327L compound heterozygous genotype is frequently observed in patients with perinatal benign HPP.^13–16^^,^^22^ Serum TNAP levels were significantly lower in *Alpl^c.1559delT/p.F327L^* mice than in WT mice. Additionally, the tibial length of *Alpl^c.1559delT/p.F327L^* mice was significantly shorter than that of WT mice. However, no marked bone hypomineralization was detected in *Alpl^c.1559delT/p.F327L^* mice. In milder HPP forms, such as perinatal benign HPP, skeletal hypomineralization is minimal, and bone deformities, such as shortening or bowing of long bones, are noted.^23^ Serum TNAP levels tended to be lower in *Alpl^c.1559delT/p.F327L^* mice than in *Alpl^+/p.R184W^* mice. Bone symptoms are considered to be associated with serum TNAP levels, and *Alpl^+/p.R184W^* mice may be less severely affected than *Alpl*^*c.1559delT/p.F327*L^ mice. Interestingly, despite exhibiting lower serum TNAP levels than *Alpl^+/p.R184W^* mice, *Alpl^c.1559delT/p.F327L^* mice showed a distinct pattern of dental abnormalities. These findings suggest that dental manifestations cannot be explained solely by circulating TNAP levels but are also influenced by the functional consequences of individual *ALPL* variants.

In the present study, we demonstrated that alveolar bone BMD is reduced in HPP model mice, indicating that alveolar bone may be particularly sensitive to reduced TNAP levels. This reduction was observed even in the absence of overt systemic skeletal osteopenia. This finding may reflect the high metabolic turnover of alveolar bone and its continuous adaptation to mechanical loading.^4^^,^^24^ Consistent with this finding, decreased OPN expression and increased CEJ–ABC distance were both pronounced in a TNAP level-dependent manner. Osteopontin is a major non-collagenous protein of mineralized tissues and a well-established marker of cementum, where it plays a critical role in cementogenesis and PDL attachment.^25^^,^^26^ Given that TNAP deficiency leads to local accumulation of inorganic pyrophosphate (PP_i_), a potent inhibitor of mineralization, both alveolar bone and cementum appear particularly susceptible to disrupted phosphate/pyrophosphate homeostasis. Enlargement of the CEJ–ABC distance suggests compromised periodontal support resulting from alveolar bone loss and/or defective cementum formation.^25^^,^^27^^,^^28^ Importantly, osteoclasts were detected at comparable levels in both WT and HPP model mice, indicating that osteoclast numbers were not significantly altered in HPP. In a high-turnover tissue, such as alveolar bone,^29^ impaired bone formation without a corresponding decrease in bone resorption would result in net bone loss. Given that TNAP deficiency impairs mineralization through PP_i_ accumulation,^1^ the reduced BMD observed in HPP model mice is likely attributable to defective mineralization rather than increased osteoclast numbers. These results suggest that an imbalance in alveolar bone remodeling may contribute to the reduced BMD observed in HPP model mice. Interestingly, despite the reduction in alveolar bone BV/TV, Tb.Th remained largely unchanged, suggesting that alveolar bone abnormalities in these HPP models may primarily reflect reduced bone volume rather than trabecular thinning.

In parallel with alveolar bone abnormalities, reduced serum TNAP levels were associated with defective dentin mineralization and formation, with additional alterations in enamel mineralization. Dentin mineralization proceeds through the deposition of hydroxyapatite crystals onto a collagen-rich extracellular matrix produced by odontoblasts and is highly sensitive to elevated PP_i_ levels.^27^^,^^30^ Enamel mineralization was also affected. Although enamel is formed by epithelial-derived ameloblasts and lacks a collagenous scaffold, its mineralization depends on tightly regulated phosphate availability, ionic transport, and pH control during the secretory and maturation stages.^31^ Reduced TNAP activity may directly impair enamel crystal growth, while insufficient dentin mineralization may secondarily disrupt ameloblast function and enamel crystal organization. Thus, enamel hypomineralization in HPP likely reflects a combination of direct and indirect effects of TNAP deficiency.

Our results suggest that dental phenotypes manifest differently according to the underlying *ALPL* variant and may not be explained solely by circulating TNAP levels. Therefore, evaluation of dental tissues may provide important information regarding the functional consequences of specific *ALPL* variants. Although radiographic evaluation of the dentition has been relatively underreported in the assessment of HPP,^32^ our findings emphasize the importance of careful morphological and radiographic examination of dental tissues.

Functionally, teeth from both HPP model groups showed increased extractability in PDL traction experiments; however, no clear difference was observed between the 2 KI groups despite their different serum TNAP levels. Tooth stability in vivo is regulated by multiple interacting factors, including occlusal loading, PDL function, eruption dynamics, and adaptive remodeling of supporting tissues. Unidirectional tensile testing evaluates resistance to simplified extraction forces and does not fully recapitulate the cyclic and multidirectional mechanical stresses experienced during mastication.^26^ Therefore, the absence of TNAP-dependent differences in tensile strength does not exclude increased susceptibility to tooth loss under physiological conditions.

Taken together, these findings indicate that oral abnormalities in HPP—including reduced alveolar bone BMD, impaired cementum formation, and defective dentin and enamel mineralization—represent primary developmental and remodeling defects intrinsic to the disease pathology rather than secondary consequences of systemic skeletal abnormalities. Cranial suture abnormalities, including craniosynostosis, are predominantly reported in severe forms of HPP.^33^ As this study focused on mild to moderate models, such features were not examined and warrant future investigation. The involvement of both periodontal and dental hard tissues highlights that HPP affects the dentoalveolar unit as a whole. Accordingly, comprehensive evaluation of the oral region, including alveolar bone and periodontal tissues, should be considered alongside systemic skeletal assessment in the diagnosis and management of HPP. Even in milder forms of the disease, dental and periodontal manifestations may precede or outweigh systemic skeletal symptoms, underscoring the importance of interdisciplinary collaboration between medical and dental specialists.^2^^,^^25^

An *Alpl* knock-out mouse was developed as a model of infantile HPP.^34^^,^^35^ However, without treatment, these model mice generally died before 2 wk of age. Formation of the mouse first molar is completed at approximately 8 wk of age.^36^ This infantile *Alpl* knock-out model is not suitable for long-term dental analysis. An A116T KI mouse model of odonto HPP has been reported.^37^ The *Alpl* c.346G>A variant, predicting an A116T substitution, was selected for KI modeling based on case reports of HPP in a European family.^38^^,^^39^ This model exhibits defective cementum formation and periodontal attachment abnormalities, which are consistent with the periodontal alterations observed in our models. However, this variant has not been reported among odonto HPP cases in Japan. Two nationwide dental surveys in Japan found no evidence of this variant in 14 and 31 cases of odonto HPP.^8^^,^^12^ The p.R184W variant was selected as a representative dominant-negative *ALPL* variant associated with odonto HPP and mild disease phenotypes. The p.R184W variant was detected in 30% of odonto HPP cases.^8^^,^^12^ Diagnoses of odonto HPP have been increasing because of the increased awareness and recognition of this disease in Japan.^6^^,^^8^^,^^12^^,^^15^ As the number of cases increases, it is desirable to develop orthodontic and implant treatments suitable for the dental manifestations of HPP.^8^ Additionally, the mouse model of odonto HPP is expected to lead to the development of treatment methods for oral manifestations. *Alpl^+/p.R184W^* KI mice will serve as a model of odonto HPP for further studies.

A limitation of this study is that we used a model in a monophyodont animal (mice have only one dentition), meaning that the major dental manifestation of early exfoliation of human primary teeth cannot be accurately reproduced. This has prompted research into other animal models, such as sheep.^40^ Another limitation is that the KI mice were analyzed on an outbred ICR genetic background. Although we initially attempted to establish the KI mice on a C57BL/6 background, the colony could not be successfully expanded. The KI mouse lines were subsequently established on an ICR background. As a result, the contribution of modifier genes cannot be completely excluded, and future studies using an inbred genetic background will be important to validate the variant-specific phenotypes observed in this study. In addition, the mice were analyzed at a relatively young age. Hypophosphatasia is a progressive metabolic bone disease with a continuous clinical spectrum rather than discrete subtypes.^4^ The mice analyzed in this study exhibited only dental symptoms in the mildest model, although bone symptoms may become apparent with aging. Furthermore, even in the mild model where bone symptoms were observed, these symptoms may potentially worsen. In addition, direct assessment of bone formation was not performed, and further studies are needed to evaluate bone formation dynamics in this model. Food intake, chewing function, and body weight beyond 12 wk of age were also not evaluated. Therefore, the functional impact of the dental phenotype on mastication and growth remains unclear and should be investigated in future studies.

ERT was approved for manufacture and marketing in Japan in 2015, ahead of the rest of the world.^41^^,^^42^ The dental symptoms of HPP vary in severity, and the number of dental visits by patients with HPP is increasing because of the availability of ERT.^8^ Patients with HPP require dental care according to disease severity. We generated KI mice harboring clinically relevant *ALPL* variants representing a range of HPP severities. Cross-breeding of these KI mice enabled us to generate mouse models of milder forms of HPP, including perinatal benign, childhood, and adult HPP. These findings suggest that variant-specific effects of *ALPL* may contribute to genotype-specific dental phenotypes that are not fully explained by circulating TNAP activity.

In conclusion, we established mouse models that recapitulate mild HPP phenotypes characterized by dentoalveolar manifestations—including alveolar bone loss, cementum defects, and dentin/enamel hypomineralization—with or without mild skeletal abnormalities. We further demonstrated that distinct dental and periodontal tissues—including alveolar bone, PDL, and tooth structures—are affected to different extents in a genotype- and phenotype-dependent manner. These mouse models provide a valuable experimental platform for investigating severity-based dental pathophysiology in HPP and for evaluating novel dental therapeutic strategies.

## Supplementary Material

Supplementary_Material_ziag147

## Data Availability

The data generated and analyzed during the current study are available from the corresponding author upon reasonable request.
