# Supplementary material for: Creation and characterization of novel knock-in mouse models of hypophosphatasia
Source: JBMR Plus. 2026 Aug 25;10(10):ziag147. doi: 10.1093/jbmrpl/ziag147 (PMC13615531; doi:10.1093/jbmrpl/ziag147)
Supplement: Supplementary file 1 [file supplementary_material_ziag147.docx]

**Supplementary Information**

Materials and methods

Generation of KI mice

A CRISPR/Cas9-mediated targeting strategy generated *Alpl* p.R184W, p.F327L, and c.1559delT KI mice. The crRNAs shown in Table S1 were designed using the online sgRNA design tool available at https://sg.idtdna.com/site/order/designtool/index/CRISPR_CUSTOM and purchased from Integrated DNA Technologies (Coralville, IA, USA). Donor ssDNAs shown in Table S1 were purchased from Eurofins Genomics (Tokyo, Japan). ICR mice were purchased from Japan SLC, Inc. (Shizuoka, Japan). The fertilized pronuclear-stage embryos were prepared by *in vivo* fertilization. Next, the ssODN (12 µM) and the complex of crRNAs (4 µM) and tracrRNA (4 µM), and Cas9 protein (0.76 mM) (Integrated DNA Technologies) in Opti-MEM (Thermo Fisher Scientific, Waltham, MA, USA) were introduced to pronuclear-stage embryos by electroporation. The embryos were then washed and cultured in a potassium simplex optimization medium (KSOM; ARK Resource, Kumamoto Japan) overnight. Approximately 200 × 2-cell embryos per line were transferred to pseudo-pregnant recipient ICR mice (CLEA Japan, Inc., Tokyo, Japan). Pups from transplanted embryos were considered as the F0 generation. The genotypes of the F0 generation were analyzed by PCR with genomic DNA extracted from the tail and primers (Table S1). Tail specimens were resuspended in 200 µL of lysis buffer containing Direct PCR Lysis Reagent Mouse Tail (Viagen Biotech, Inc., Los Angeles, CA, USA) and 10 mg/ml Proteinase K (Promega K.K., Tokyo, Japan). After incubation at 55 °C overnight, followed by incubation at 95 °C for 10 min, the samples were spun down and kept at 4 °C. The PCR cycle was subjected to an initial denaturation at 95 °C for 2 min, followed by 35 cycles of 95 °C (30 s), 58 °C (30 s), 72 °C (30 s), and a final extension at 72 °C (7 min). PCR products were electrophoresed on a 2% agarose gel, and DNA was visualized by ethidium bromide staining. PCR was conducted using the primers shown in Table S1 to validate the CRISPR editing by Sanger sequencing. The resulting PCR products were purified or cloned into the pGEM-T Easy vector (Promega K.K.) by TA cloning, and Sanger sequencing analysis was performed to determine the DNA sequence of the edited mice. One or two founder (F0) mice, including the KI allele, were mated with WT mice to obtain an F1 generation.

Those with a c.1559delT or p.F327L type variant were crossed with heterozygous KI mice carrying the same or different variants, and also heterozygous KI male mice with a p.R184W type and WT female mice. Tail samples were obtained from offspring at the age of 3 weeks, and genomic DNA was extracted for genotyping.

**Supplementary Figure 1. Representative PCR-based genotyping of *Alpl* knock-in (KI) and wild type (WT) mice.**

(A) Genotyping of WT mice.
(B) Genotyping of heterozygous mice (*Alpl^+/p.R184W^*).

(C) Genotyping of compound heterozygous mice (*Alpl^c.1559delT/p.F327L^*).
Expected band sizes (bp) are indicated. DNA was extracted from tail biopsies, and PCR products were separated by agarose gel electrophoresis.

**Supplementary Figure 2. Schematic illustration of the measurement sites and analytical methods for the first molar on a sagittal section at the level of the maximum mesiodistal width.**

(A) Measurement sites for mineral density (MD). Open circles indicate the enamel measurement site, and the filled circle indicates the dentin measurement site.
(B) Measurement of enamel and dentin widths. The solid double-headed arrow indicates enamel width, and the dashed double-headed arrow indicates dentin width. E, enamel; D, dentin.
(C–E) Procedure for calculating the pulp cavity area. (C) Sagittal section of the first molar at the level of the maximum mesiodistal width. (D) Isolated image of the tooth shown in (C). (E) Segmented image of the pulp cavity extracted from (D).

**Supplementary Figure 3. Periodontal ligament traction test with tooth extraction.**

(A–E) Images of the periodontal ligament traction test. The maximum load required to extract a tooth was measured during traction of the mandibular first molar in 12-week-old mice.

(F) Extracted mandibular first molar.

(G) Tooth sockets following complete dislocation of the mandibular first molar.

**Supplementary Figure 4. Serum TNAP activity, body weight, tibial morphology, and bone mineral density in female wild type (WT) and knock-in (KI) mice.**

(A) Serum TNAP activity. (B) Body weight. (C) Tibial length. (D) Tibial width. (E–H) Trabecular and cortical bone mineral density (BMD) of the femur and tibia. Statistical analysis was performed using one-way ANOVA followed by Dunnett’s multiple comparisons test. Data are presented as mean ± SEM. Sample sizes are indicated in each graph. * *p* < 0.05, ** *p* < 0.01.

 **Supplementary Figure 5. Phenotypic characterization of heterozygous *Alpl* mutant mice.**

(A) Body weight, (B) tibial length, and (C) tibial width in male *Alpl^+/c.1559delT^* (n = 3–4) and *Alpl^+/p.F327L^* (n = 7) mice compared with wild type controls (n = 15). Statistical analysis was performed using one-way ANOVA followed by Dunnett’s multiple comparisons test. No statistically significant differences were identified between heterozygous mice and wild type mice. Data are presented as mean ± SEM.

**Supplementary Figure 6. Micro-computed tomography analysis of trabecular bone microarchitecture in wild type (WT) and knock-in (KI) mice.**

(A–F) Trabecular microarchitectural parameters, including bone volume fraction (BV/TV) and trabecular thickness (Tb.Th), of the femur, tibia, and alveolar bone in WT and KI mice. Statistical analysis was performed using one-way ANOVA followed by Dunnett’s multiple comparisons test. Data are presented as mean ± SEM (n = 5 males per group). ** *p* < 0.01.

**Supplementary Figure 7. Micro-computed tomography analysis of incisors in wild type (WT) and knock-in (KI) mice.**

Representative coronal sections of the mandibular incisors at the level of the mesial aspect of the first molar.

(A) WT mice; (B) *Alpl^+/p.R184W^* mice; and (C) *Alpl^c.1559delT/p.F327L^* mice. Arrowheads: pulp chamber. D, dentin; E, enamel.

**Supplementary Figure 8. Sex-stratified analysis of dental parameters in wild type (WT) and knock-in (KI) mice.**

(A–E) Male (WT: n = 9–15, *Alpl^+/p.R184W^*: n = 7–8, *Alpl^c.1559delT/p.F327L^*: n = 4) and (F–J) female (WT: n = 10–11, *Alpl^+/p.R184W^*: n = 8–9, *Alpl^c.1559delT/p.F327L^*: n = 7–8) mice.

(A, F) Enamel width and (B, G) dentin width.
(C, H) Pulp/tooth ratio of the first molar.

(D, I) Mineral density (MD) of enamel and (E, J) dentin.
Statistical analysis was performed using one-way ANOVA followed by Bonferroni’s multiple comparisons test. Individual data points are shown with mean ± SEM. Similar genotype-related trends were observed in male and female mice, with no apparent sex-specific differences.

* *p* < 0.05, ** *p* < 0.01 and **** *p* < 0.0001.

**Supplementary Figure 9.** **Sex-stratified analysis of periodontal parameters in wild type (WT) and knock-in (KI) mice.**

(A, B) Male (n = 2 per genotype) and female (n = 3 per genotype) mice.
(A) Alveolar bone loss was measured at the molar from the cemento–enamel junction (CEJ) to the alveolar bone crest (ABC).
(B) Periodontal ligament traction test with tooth extraction.
Individual data points are shown with mean ± SD. Blue and red symbols indicate male and female mice, respectively. Although the sample size was limited, no consistent sex-specific trend was observed across any parameter.

**Supplementary Figure 10. Histological analyses of incisors of wild type (WT) and knock-in (KI) mice.**

H-E staining (A–C) and immunohistochemical analysis of osteopontin (D–I) in WT and KI mice. Representative cross-sections of the mandibular incisors are shown for: (A, D, G) WT mice; (B, E, H) *Alpl^+/p.R184W^* mice; and (C, F, I) *Alpl^c.1559delT/p.F327L^* mice. G–I are higher magnification images of the squares in D–F. Scale bars: (A–F) 200 µm; (G–I) 20 µm. Arrow: disturbed cementum formation, arrowheads: osteopontin. C, cementum; D, dentin.

**Table S1. Nucleotide sequences used for the experiments**

| Knock-in | Name of material | Sequence |
| --- | --- | --- |
| c.1559delT | crRNAs | 5′-TCCCTACGCACCCTGTTCTG-3′ |
|  | ssODN | 5′-CACAAAAGGAGGGGAGCTGGCTGTCCATTGCGGGCTTGTGGGACCTGCACCCTCAGAACAGGGTGCGT(A)GGGAGAGCACAGCCAGTGGAAGCAGCAGGGCCCCTGGGGAGGGTGCGCTCCCAGAGCCGGCCCAGGG-3′ |
|  | Genotyping primer forward for WT | 5′-CCTCAGAACAGGGTGCATA-3′ |
|  | Genotyping primer forward for KI | 5′-CCTCAGAACAGGGTGCATG-3′ |
|  | Genotyping primer reverse | 5′-TGACCTACAGCTCACAACAAC-3′ |
|  | PCR for Sanger sequence forward | 5′-GGAAGTTGCCTGGACCTCTC-3′ |
|  | PCR for Sanger sequence reverse | 5′-TGACCTACAGCTCACAACAAC-3′ |
| p.F327L | crRNAs | 5′-CCTCAAAGGCTTCTTCTTGC-3′ |
|  | ssODN | 5′-GCCTAACCCACGTCATGTTTCTGGAAGGGTTGTCTCC  ACCACTTCCTACCTTCCACCAGCAAGAAGAGGCCTTTGAGGTTTTTGGTCAGGATCCGGAGGGCCACCTCCACCATCTCGGAGAGCGAAGGGTCAGTC-3′ |
|  | Genotyping primer forward for WT | 5′-CCTTCCACCAGCAAGAATAA-3′ |
|  | Genotyping primer forward for KI | 5′-CCTTCCACCAGCAAGAATAG-3′ |
|  | Genotyping primer reverse | 5′-GATCCACAGAGCTTCCCAGG-3′ |
|  | PCR for Sanger sequence forward | 5′-CCCCTCTCCTCTGAATTCCC-3′ |
|  | PCR for Sanger sequence reverse | 5′-GATCCACAGAGCTTCCCAGG-3′ |
| p.R184W | crRNAs | 5′-CACACTCGGCCGATCGGGAC-3′ |
|  | ssODN | 5′-GCGATGTCCTTGCAGCCCTGGCTCAGAGCCTCTGGTG  GCATCTCGTTATCCGAGTACCAGTCCCAATCGGCCGAGTGTGCGTAGGCTGCACTGGGTGTGGCGTGGTTCACCCGAGTGGTAGTCACAATGCCCACG-3′ |
|  | Genotyping primer forward for WT | 5′-TTATCCGAGTACCAGTCTCG-3′ |
|  | Genotyping primer forward for KI | 5′-TTATCCGAGTACCAGTCTCA-3′ |
|  | Genotyping primer reverse | 5′-CTCTCCACTTGACGTCCCTT-3′ |
|  | PCR for Sanger sequence forward | 5′-CTGCCTGTTCCAACCACAAA-3′ |
|  | PCR for Sanger sequence reverse | 5′-CTCTCCACTTGACGTCCCTT-3′ |

KI: knock-in, WT: wild type
